# Odontomas are associated with impacted permanent teeth in orthodontic patients

**DOI:** 10.4317/jced.56101

**Published:** 2019-09-01

**Authors:** Vanessa-Silvestre-de Aquino da Silva, Renato-do Prado-Gomes Pedreira, Felipe-Fornias Sperandio, Denismar-Alves Nogueira, Marina-Lara de Carli, João-Adolfo-Costa Hanemann

**Affiliations:** 1 School of Dentistry, Department of Clinic and Surgery, Federal University of Alfenas, Alfenas, MG, Brazil; 2 Marcelo Pedreira Orthodontic Institute, Alfenas, MG, Brazil; 3 Institute of Biomedical Sciences, Department of Pathology and Parasitology, Federal University of Alfenas, Alfenas, MG, Brazil; 4 Institute of Exact Sciences, Department of Statistics, Federal University of Alfenas, Alfenas, MG, Brazil

## Abstract

**Background:**

Odontomas are the most frequent odontogenic tumors in the oral cavity and can result in failure of eruption of permanent teeth or be associated with impacted teeth.

**Material and Methods:**

The present study evaluated the prevalence of complex and compound odontomas in non-syndromic patients prior to the onset of orthodontic treatment. Panoramic radiographs of 4,267 non-syndromic patients were evaluated; 22 cases were included being 54.5% complex and 45.4% compound odontomas.

**Results:**

The sample was composed predominantly by White males with mean age of 14.5 years. Complex odontomas were commonly found in the maxilla (83.3%) while compound type was mostly located on mandible (60%), presenting a significant association (*P*=0.027). Moreover, odontomas were significantly associated with impacted teeth (*P*<0.0001). The most frequently odontoma-associated impacted teeth were lower canines, followed by upper central incisors and upper canines, while impacted teeth with no odontoma were predominantly upper canines, lower second premolars and upper second premolars. Compound and complex odontomas showed mean size of 10.5 and 7.25 mm, respectively, presenting significant association between lesion size and odontoma type (*P*=0.021).

**Conclusions:**

Odontomas affected mainly White male patients with mean age of 14.5 years, being the complex type commonly found in the maxilla and the compound type mostly located on mandible. Furthermore, odontomas were significantly associated with impacted teeth, affecting mainly lower canines. Early diagnosis and correct treatment are essential to avoid any complications, such as prolonged retention of primary teeth and delayed eruption of permanent teeth.

** Key words:**Odontoma, odontogenic tumors, dental anomalies.

## Introduction

Odontomas are the most frequent odontogenic tumors in the oral cavity (1), corresponding to 21 to 67% of these tumors (2), although the last World Health Organization Classification of Head and Neck Tumors published in 2017 (3) considers these lesions as hamartomas or tumour-like malformations composed of dental hard and soft tissues. Odontomas are classified into compound or complex types; the first is characterized by radiopaque mass that resemble to denticles surrounded by a thin soft tissue capsule, while the latter consists of a disordered mass of calcified tissue also bordered by thin soft tissue component (3). The compound type is more frequent than complex, showing no predilection for gender, age or location (1). Interestingly, compound odontomas are diagnosed earlier than the complex type, probably due to involvement of upper anterior permanent teeth in most cases (1).

The pathogenesis of the lesion remains unknown; nevertheless, different etiopathogeneses have been proposed for each type of odontoma: complex type could be derived from the terminal stage of maturation of an ameloblastic fibroma developing during childhood or an ameloblastic fibro-odontoma, comprising an hamartomatous origin that differs from the neoplastic line of ameloblastic fibroma (4). On the other hand, the compound odontomas are considered a malformation, probably due to a locally conditioned hyperactivity of the dental lamina (4).

Odontomas are commonly diagnosed in routine radiographs, in the presence of retained primary tooth or due to failure of eruption of permanent teeth (1,5,6). In cases associated with impacted teeth, the tumor generally is located on the eruption pathway of permanent teeth, avoiding the normal eruption of the related teeth (6). Consequently, impacted teeth can contribute to develop malocclusion Class III (7). In addition, long-standing lesions can cause limitation of mouth opening, enlargement of the buccal and lingual cortical plates resulting in facial asymmetry, or painful symptoms (8,9).

Nevertheless, there is no agreement regarding the epidemiological and clinical features of odontomas (1). Considering that the lesion is asymptomatic in most cases and may result in delayed eruption of permanent teeth and prolonged retention of deciduous teeth, it is extremely important to know the prevalence and characteristics of odontomas in patients seeking orthodontic treatment. Thus, the aim of the present study was to evaluate the prevalence of complex and compound odontomas in non-syndromic patients prior to the onset of orthodontic treatment.

## Material and Methods

Orthodontic pretreatment panoramic radiographs of 4.267 non-syndromic patients were evaluated between 1998 and 2018. Inclusion criteria were good quality radiographs and presence of complex or compound odontoma, remaining 22 cases, among which 10 (45.6%) were females and 12 (54.5%) were males. The analysis of panoramic radiographs was performed by a single examiner using a negatoscope in ideal lighting conditions; for the included cases, tumor location and affected teeth, odontoma type and lesion size were obtained. Clinical data on gender, age and race were extracted from the records.

Informed consent forms were obtained from patients or their guardians prior to orthodontic treatment. The study was analyzed and approved by the Research Ethics Committee of our institution (protocol number 2.487.661). The authors read the Helsinki Declaration and followed the guidelines in this investigation.

Odontoma radiographic diagnostic criteria were based on Philipsen *et al.* (4) and World Health Organization (WHO) Classification of Head and Neck Tumours (3) as follows: tumour-like malformations (hamartomas) composed of dental hard and soft tissues presenting a well-demarcated radiopacity bordered by a thin soft tissue capsule and an adjacent corticated layer of bone, being (1) Complex type: the lesion shows an amorphous, solitary, disorganized mass of calcified tissue; (2) Compound type: the lesion shows numerous tooth-like radiopaque structures.

Impacted teeth associated with an odontoma were also investigated according the following criterion: the teeth were considered impacted when other teeth, bone or soft tissues interfered with their eruption, in normal functional occlusion (10).

The collected data were analyzed using the IBM SPSS v20 (Statistical Package for the Social Sciences) software (11). Statistical analyses were performed using Fisherʼs exact test and Mann–Whitney test, employing a significance level of 5%.

## Results

The sample was composed by 12 (54.55%) complex and 10 (45.45%) compound odontomas. Age ranged from 9 to 41 years, with mean age of 14.5 years, being 12.45 and 17.1 years for the complex and compound types, respectively, with no significant correlation between age and odontoma type (*P*=0.172). Concerning race, 13 (59.1%) patients were White and 9 (40.1%) were Brown. Male patients were slightly more prevalent than female; among men, 6 (50%) presented with complex odontomas and 6 (50%) with the compound type. Six (60%) women presented complex odontomas and 4 (40%) showed the compound type. There was no significant correlation between gender and odontoma type (*P*=0.69).

Tumor location revealed prevalence for maxilla, affecting 13 (59.1%) patients ( Table 1). Interestingly, complex odontomas were commonly found in maxilla (83.3%) while compound type was mostly located on mandible (60%), presenting a significant association (*P*=0.027) ( Table 1). Compound odontomas were related to an impacted tooth in 7 (70%) cases, while complex odontomas exhibited this relationship in just 6 (50%) patients; there is no significant correlation between them (*P*=0.415) ( Table 2).

**Table 1:**
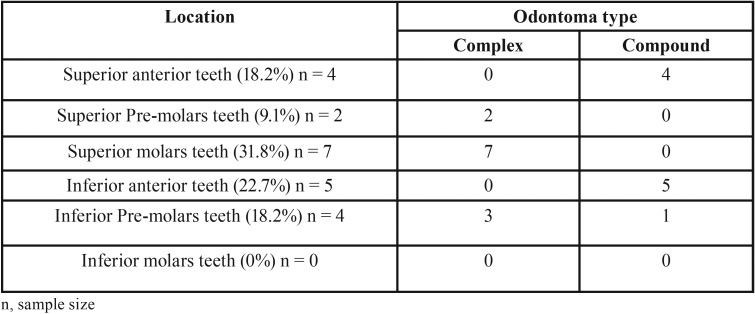
Distribution of odontomas according to location.

**Table 2:**
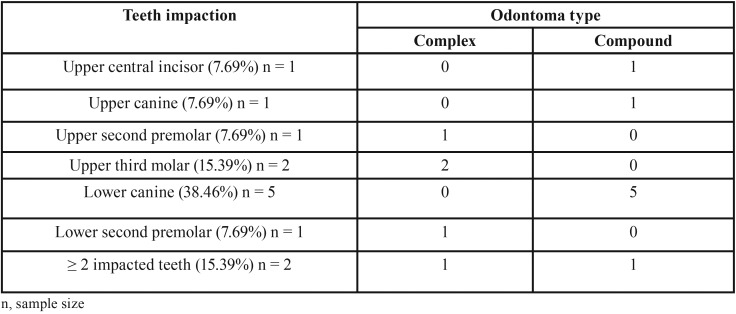
Frequency of impacted teeth associated with odontoma.

Nevertheless, odontomas were significantly associated with impacted teeth, being 13 (59.1%) cases with any impacted teeth related to the lesion, while 248 (5.84%) patients without odontomas presented any impacted teeth (*P*<0.0001). The most frequently odontoma-associated impacted teeth were lower canines (38.46%), followed by upper central incisors and upper canines, as described on  Table 2, while impacted teeth with no odontoma were upper canine (48.85%), lower second premolar (17.56%), upper second premolar (11.45%), lower canine (6.88%), lower second molar (5.35%), lower first premolar (3.05%), upper central incisor (3.05%), upper lateral incisor (1.53%), upper first premolar (0.76%), upper second molar (0.76%) and lower first molar (0.76%).

The lesion size ranged from 4 to 17 mm, with mean size of 8.73 mm; compound and complex odontomas showed mean size of 10.5 and 7.25 mm, respectively, presenting significant association between lesion size and odontoma type (*P*=0.021).

## Discussion

Odontomas are generally diagnosed on panoramic radiographs, which is a proper technique for diagnosis (12,13). Once most lesions are found on routine radiographs (1,14), clinical examination and panoramic radiographies previous to every dental treatment are crucial to detect an odontoma, avoiding prolonged retention of primary teeth, delayed eruption of permanent teeth and possible space loss (15).

These lesions are commonly found in young adults, with a mean age of 26 years, being greater for the complex type (1,6,13); nevertheless, our results showed mean age of 14 years old, reflecting the studied population, which was composed by orthodontic pretreatment patients. Men were slightly more affected than women, similar to Da Silva *et al.* (6) and Hidalgo-Sanchez *et al.* (1), although other authors reported female predominance (13). These divergent results can be attributed to ethnic differences as well as to the inclusion criteria used in each study. Patient age and gender did not show any correlation with the odontoma type, similar to previous studies (6,13); confirming that there is equal distribution of compound and complex odontomas concerning age and gender.

Compound odontomas exhibit great predilection for the anterior maxilla (6,15), corroborating with the theory proposed by Philipsen *et al.* (4) that this region presents conditioned hyperactivity of the dental lamina favoring the development of compound type and supernumerary teeth. However, our results showed prevalence for anterior mandible with significant differences between compound and complex type (*P*=0.027), evidencing that anterior inferior region could also present the conditioned hyperactivity of the dental lamina. Some authors have reported odontoma in anterior mandible (5).

On the other hand, complex odontomas were predominantly found in the posterior region of the maxilla, presenting significant lower size than the compound type. The distribution of complex odontomas has presented great variability, from preponderance for posterior mandible (6,13) to anterior maxilla (16). Odontomas are generally smaller than the adjacent teeth, but complex type can reach large size and cause facial asymmetry, expansion of the buccal and lingual cortical plates, impacted teeth and limitation of mouth opening (8).

Concerning tooth impaction, 59% of odontomas were associated with an impacted tooth, similar to Dunfee *et al.* (17), showing predilection for lower canine. In fact, most of the impacted teeth associated to an odontoma are located on anterior region of the maxilla or mandible (6). Impacted permanent incisors are generally associated to compound odontoma rather than complex type (18). Interestingly, our results showed significant association between odontoma and impacted teeth (*P*<0.0001), emphasizing the importance of early diagnosis of the lesion and proper treatment.

Treatment for an odontoma involves surgical excision via an intraoral approach upon general or local anesthesia (13), with no reported recurrence up to 20 years (6). Cases that present any malocclusion may need complementary orthodontic treatmen t(14). Early removal of an odontoma may result in spontaneous eruption of the impacted teeth if incomplete root development is seen (19).

In conclusion, odontomas were more prevalent in White male patients with mean age of 14.5 years. Complex odontomas were commonly found in the maxilla while compound type was mostly located on the mandible. Furthermore, odontomas were significantly associated with impacted teeth, affecting mainly lower canines. Early diagnosis and correct treatment are essential to avoid any complications, such as prolonged retention of primary teeth and delayed eruption of permanent teeth.
